# Protectin DX ameliorates palmitate- or high-fat diet-induced insulin resistance and inflammation through an AMPK-PPARα-dependent pathway in mice

**DOI:** 10.1038/s41598-017-01603-9

**Published:** 2017-05-03

**Authors:** Tae Woo Jung, Hyoung-Chun Kim, A. M. Abd El-Aty, Ji Hoon Jeong

**Affiliations:** 1 Research Administration Team, Seoul National University Bundang Hospital, Gyeonggi, Republic of Korea; 2 Neuropsychopharmacology and Toxicology Program, College of Pharmacy, Kangwon National University, Chunchon, Republic of Korea; 3 Department of Veterinary Pharmacology and Toxicology, College of Veterinary Medicine, Konkuk University, Seoul, Republic of Korea; 4 Faculty of Veterinary Medicine, Cairo University, 12211 Giza, Egypt; 5 Department of Pharmacology, College of Medicine, Chung-Ang University, Seoul, Republic of Korea

## Abstract

Protectin DX (PDX), a double lipoxygenase derivative of docosahexaenoic acid, has been reported to attenuate inflammation and insulin resistance. In the current study, we explored the effects of PDX on hyperlipidemia-induced insulin resistance and inflammation through AMP-activated protein kinase (AMPK) and peroxisome proliferator-activated receptor α (PPARα). PDX attenuated the impairment of insulin receptor substrate 1/Akt–mediated insulin signaling in palmitate-treated differentiated C2C12 cells and soleus skeletal muscle of HFD-fed mice. Furthermore, PDX treatment significantly ameliorated HFD-induced weight gain and improved glucose tolerance in mice. Nuclear factor kB nuclear translocation, inhibitory kBα phosphorylation, and expression of proinflammatory cytokines were markedly attenuated by PDX in both *in vitro* and *in vivo* models. PDX treatment markedly augmented AMPK phosphorylation and PPARα expression in C2C12 cells and in skeletal muscle of mice. AMPK- and PPARα-specific siRNAs significantly abrogated the suppressive effects of PDX on palmitate-induced insulin resistance and inflammation. Furthermore, PDX markedly stimulated the expression of genes related to fatty acid oxidation. These effects of PDX were significantly suppressed by AMPK and PPARα siRNAs. In conclusion, our results demonstrate that PDX ameliorates insulin resistance and inflammation and stimulates fatty acid oxidation through AMPK- and PPARα-mediated pathways in skeletal muscle.

## Introduction

Physical activity has beneficial effects on metabolic syndrome, including insulin sensitivity, in humans^1, 2^. Therefore, screening for substances that are able to mimic exercise by inducing interleukin-6 (IL-6) expression is important for the treatment of metabolic diseases. White *et al*. demonstrated that protectin DX (PDX), a product of sequential lipoxygenation of docosahexaenoic acid (DHA)^3, 4^ induces IL-6 in skeletal muscle, leading to attenuation of insulin resistance and hepatic gluconeogenesis in mice through signal transducer and activator of transcription 3 (STAT3)-dependent pathway. Moreover, this is associated with a decrease in inducible nitric oxide synthase (iNOS) and c-Jun N-terminal kinase (JNK) in the liver and skeletal muscle, and amelioration of lipid-induced inflammation in macrophages^5^. However, direct effects of PDX on insulin resistance and inflammation, and its underlying mechanisms in skeletal muscle remain unclear.

Elevated levels of serum non-esterified fatty acid (NEFA), also called free fatty acid (FFA), are observed in insulin-resistant humans^6^. Saturated NEFA induces defects in insulin signaling^7^. Furthermore, saturated FFA causes inflammation, which leads to insulin resistance through several pathways associated with iNOS^8^, JNK^9–11^, p38 mitogen-activated protein kinase (MAPK)^11^, protein kinase C (PKC)^12^, or toll-like receptors (TLRs)^10^. Among these, PKC- and TLR-mediated pathways lead to activation of nuclear factor-kB (NFkB), one of the most important proinflammatory regulators, resulting in impaired insulin signaling in skeletal muscle^13^.

In this study we investigated the effects of PDX on obesity-associated inflammation and insulin resistance. Furthermore, we have further elucidated the cellular mechanisms by which PDX affects insulin resistance and inflammation by exploring downstream signal transduction associated with AMP-activated protein kinase (AMPK) and peroxisome proliferator-activated receptor (PPAR) α in differentiated C2C12 cells and skeletal muscle of mice.

## Results

### PDX treatment attenuates hyperlipidemia-induced insulin resistance in differentiated C2C12 cells and skeletal muscle of mice

**Figure 1:**
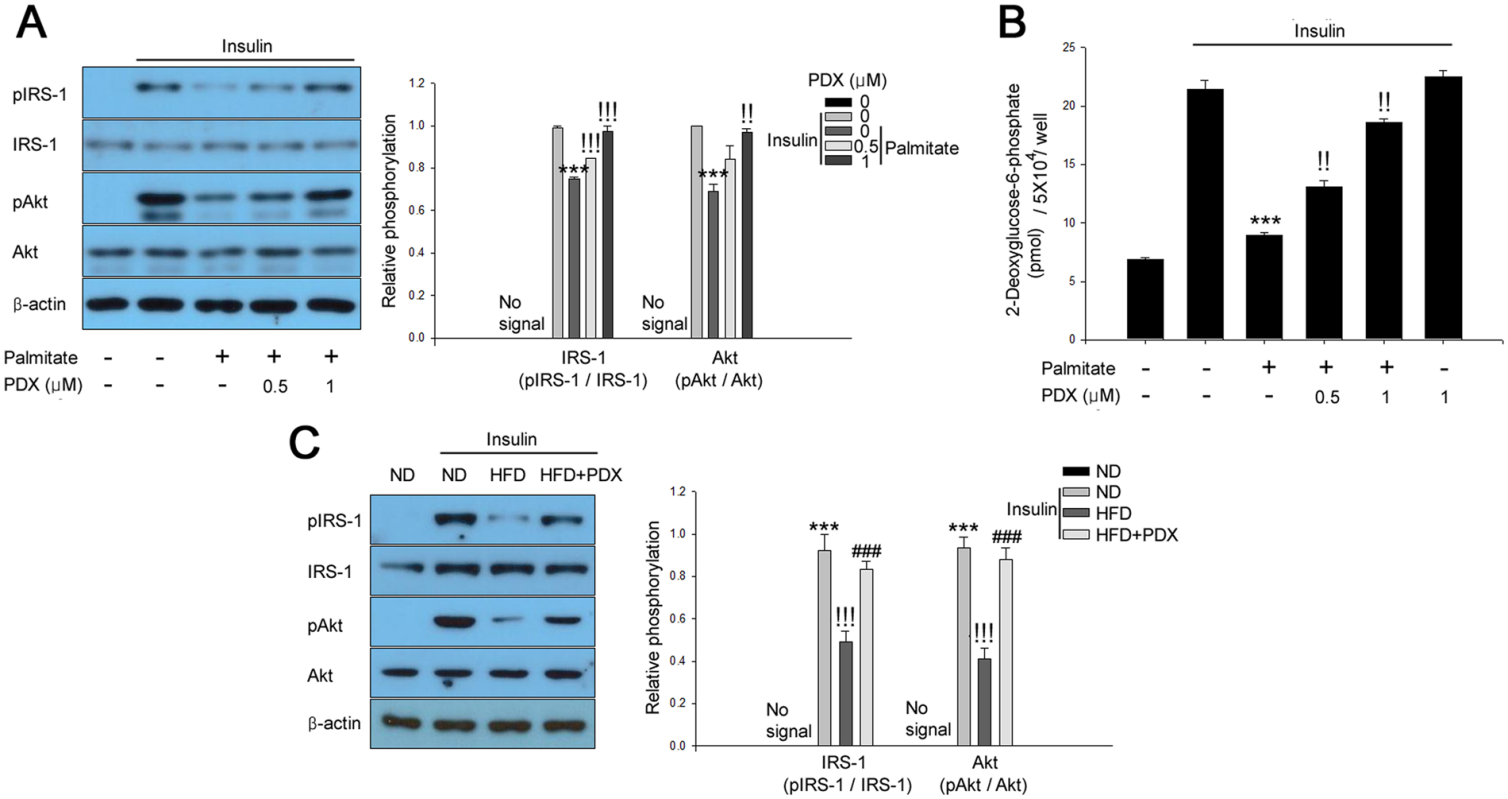
PDX improves insulin resistance in differentiated C2C12 cells and skeletal muscle of mice. (**A**) Western blot analysis of phosphorylation of IRS-1 and Akt and (**B**) 2-deoxyglucose uptake in C2C12 myocytes in the presence of 200 μM palmitate and PDX (0–1 μM) for 24 hr. Human Insulin (10 nM) stimulates IRS-1 and Akt for 3 min. (**C**) Western blot analysis of IRS-1 and Akt phosphorylation in soleus skeletal muscle from HFD-fed mice treated with PDX (five animals per treatment group). Means ± SEM were calculated from three independent experiments. ^***^ *P* < 0.001 when compared to levels in control or ND treatment. ^!!!^ *P* < 0.001 and ^!!^ *P* < 0.01 when compared to palmitate or insulin injected ND treatment. ^###^ *P* < 0.001 when compared to insulin-injected HFD treatment.

To evaluate the effects of PDX on palmitate-induced insulin resistance, we investigated the effect of PDX on levels of insulin-stimulated Akt and IRS-1 phosphorylation and glucose uptake. With reference to a previous report^5^, differentiated C2C12 mouse skeletal myocytes were treated with 0–1 μM PDX for 24 hr. Impaired insulin signaling was observed in C2C12 cells treated with palmitate (Fig. 1A and B) and in isolated soleus skeletal muscle of HFD-fed mice (Fig. 1C). However, PDX administration markedly abrogated these affects *in vitro* and *in vivo*.

### PDX attenuates HFD-induced insulin resistance in mice

**Figure 2:**
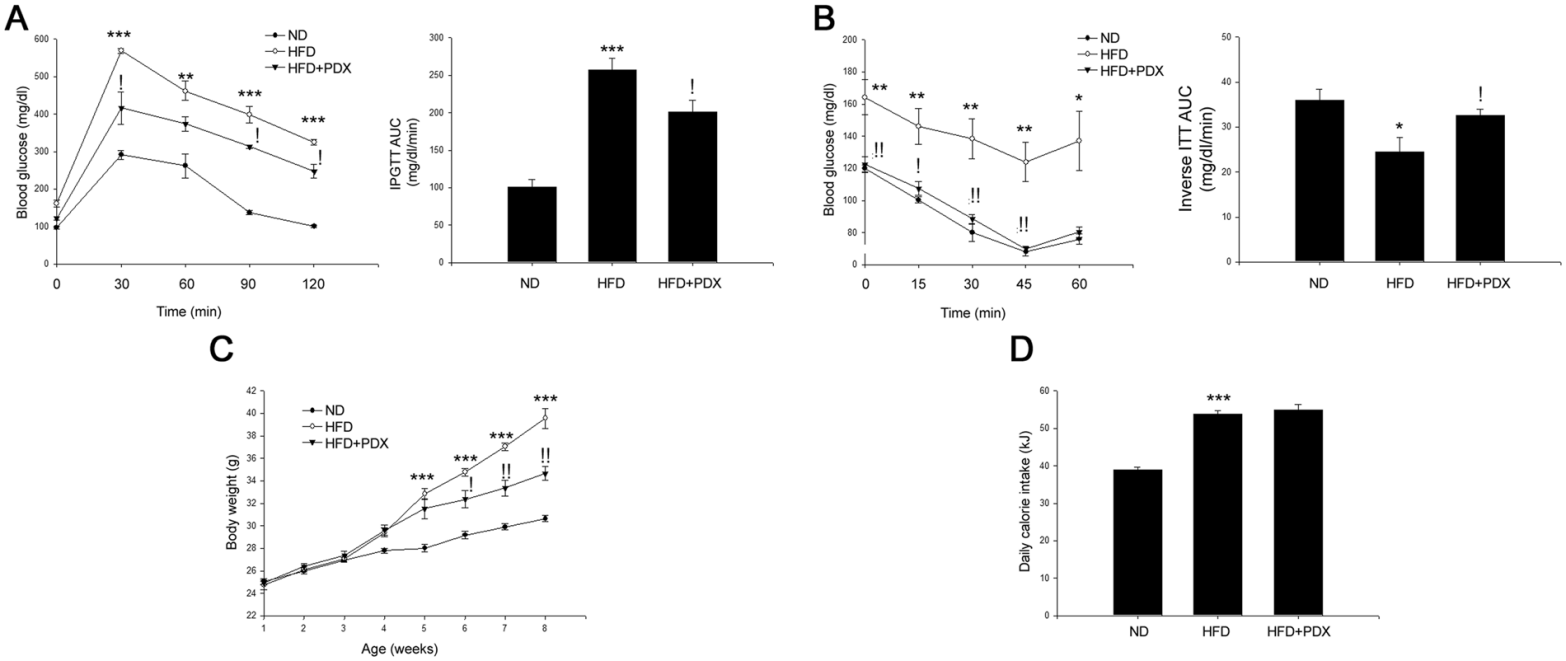
PDX treatment attenuates insulin resistance in mice. (**A**) IPGTT of experimental mice and the glucose area under the curve (AUC) during IPGTT. (**B**) ITT and the glucose inverse AUC during ITT. (**C**) Body weight measurement and (**D**) daily energy intake in mice (five animals per treatment group). Closed circles, ND; opened circles, HFD; closed triangles, HFD + PDX. Means ± SEM were calculated data obtained from five separated animals. ^***^ *P* < 0.001 and ^**^ *P* < 0.01 when compared to the ND treatment. ^!!^ *P* < 0.01 and ^!^ *P* < 0.05 when compared to the HFD.

We next examined the effects of PDX on glucose tolerance and insulin tolerance by performing IPGTTs and ITTs. Test results showed that HFD markedly aggravated glucose tolerance and insulin sensitivity compared with ND; however, PDX treatment significantly reversed these changes (Fig. 2A and B). PDX treatment did not influence calorie intake, although it markedly reduced the HFD-induced increase in body weight (Fig. 2C and D). Furthermore, PDX treatment significantly reduced the weight of epididymal adipose tissue and liver in HFD-fed mice (Supplemental Figure S1).

### PDX ameliorates palmitate-induced inflammation in differentiated C2C12 cells and soleus skeletal muscle of HFD-fed mice

**Figure 3:**
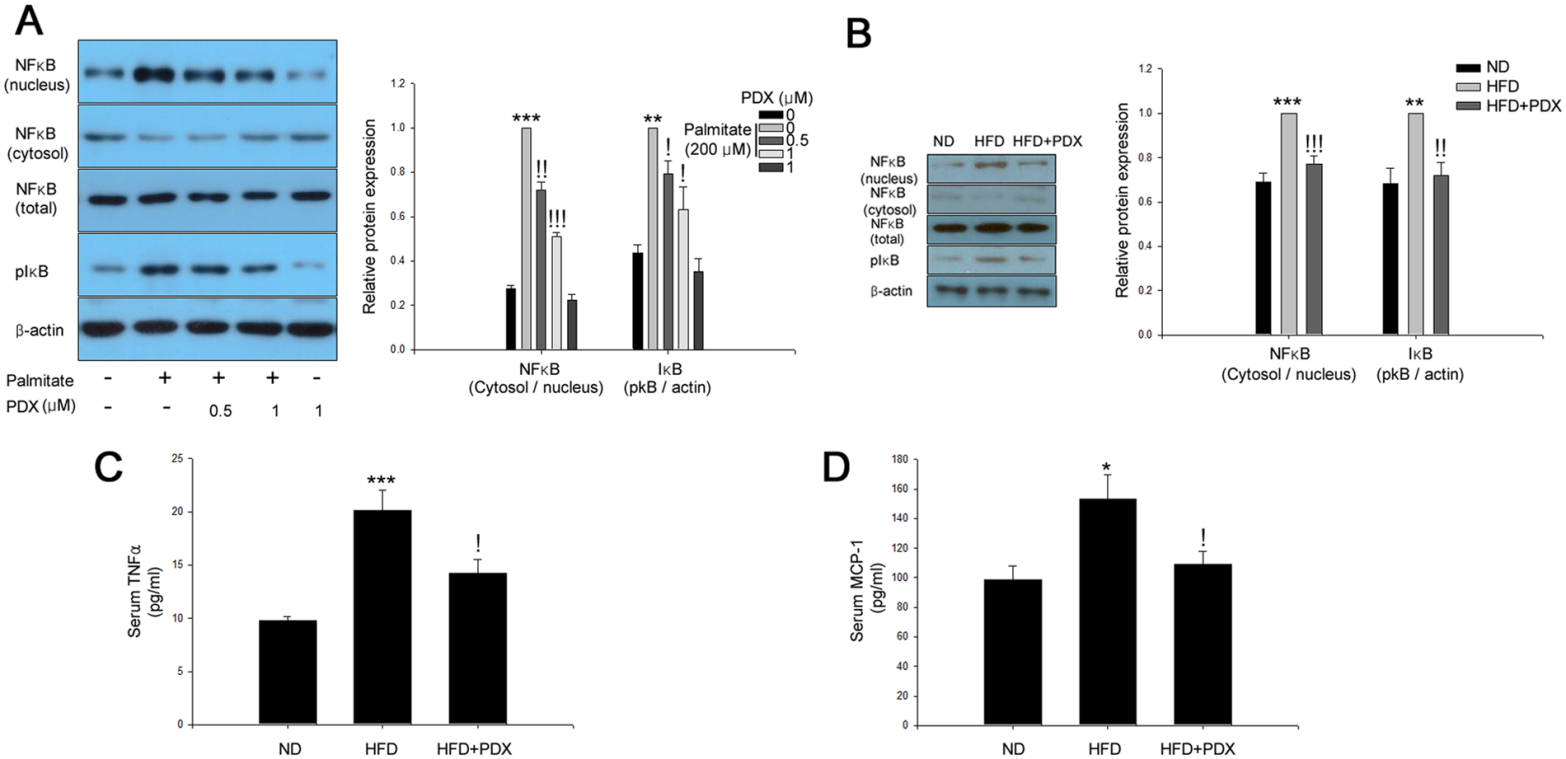
PDX ameliorates inflammation in skeletal muscle cells. (**A**) Western blot analysis of palmitate-induced NFkB nuclear translocation and IkB phosphorylation in differentiated C2C12 cells treated with 200 μM palmitate and PDX (0–1 μM) for 24 hr. (**B**) Western blot analysis of HFD-induced NFkB nuclear translocation and IkB phosphorylation in soleus skeletal muscle of mice treated with PDX (1 μg/mouse/day) for 8 weeks. Serum analysis of (**C**) TNFα and (**D**) MCP-1 of mice treated with HFD and PDX (five animals per treatment group). Means ± SEM were calculated data obtained from five separated animals. ^***^ *P* < 0.001 and ^**^ *P* < 0.01 when compared to the control or the ND treatment. ^!!!^ *P* < 0.001, ^!!^ *P* < 0.01, and ^!^ *P* < 0.05 when compared to levels in the palmitate or the HFD treatment.

PDX alleviated palmitate-induced nuclear translocation of NFkB and phosphorylation of inhibitory kBα (IkBα) in C2C12 cells in a dose-dependent manner (Fig. 3A). As shown in Fig. 3B, PDX administration also significantly suppressed HFD-induced NFkB nuclear translocation and IkBα phosphorylation in soleus skeletal muscle of mice (Fig. 3B). The HFD-induced upregulation of serum tumor necrosis factor alpha (TNFα) and monocyte chemotactic factor-1 (MCP-1) expression was also reduced by PDX treatment (Fig. 3C and D).

### PDX prevents palmitate-induced inflammation leading to attenuation of insulin resistance *via* an AMPK-dependent pathway

**Figure 4:**
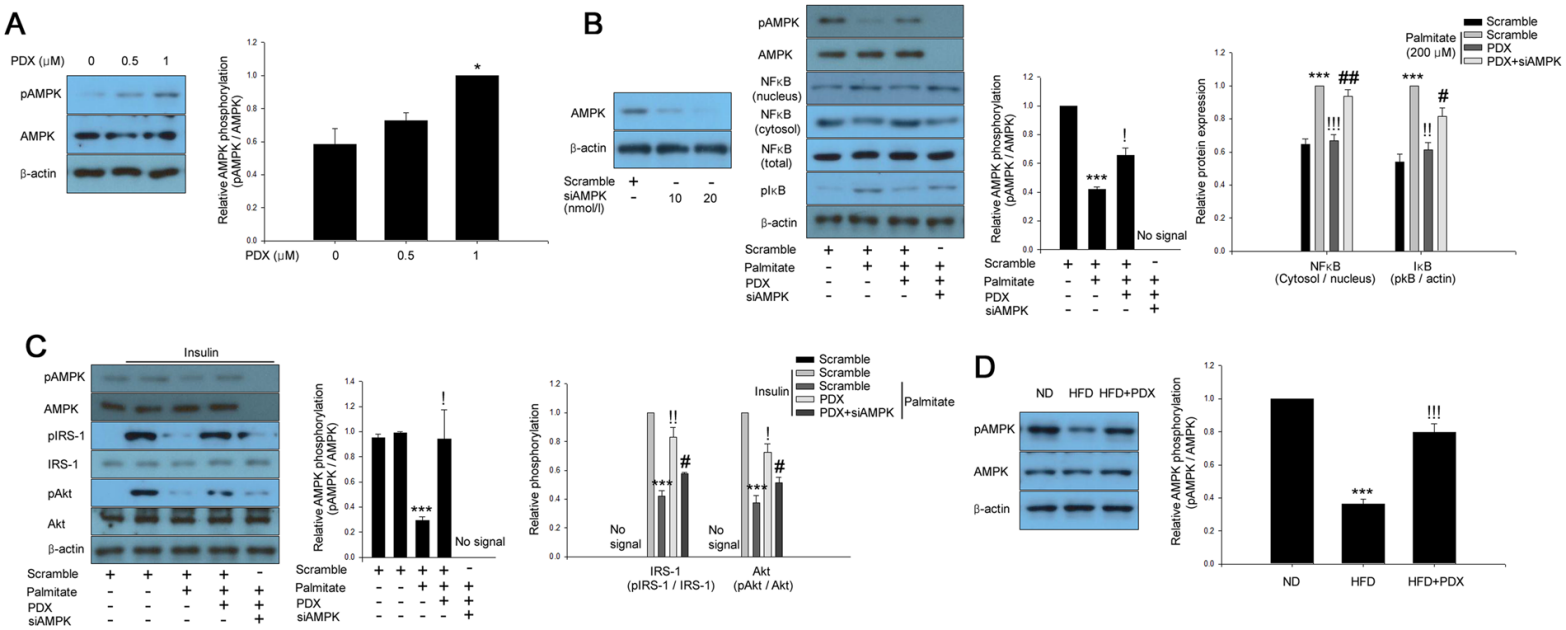
PDX attenuates inflammation and insulin resistance via an AMPK-dependent pathway. (**A**) Western blot analysis of AMPK phosphorylation in differentiated C2C12 cells (0–1 μM) for 24 hr. (**B**) Confirmation of AMPK siRNA efficiency differentiated C2C12 cells. Western blot analysis of AMPK phosphorylation and palmitate (200 μM)-induced inflammation markers in AMPK siRNA (20 nM)-transfected differentiated C2C12 cells treated with PDX (0–1 μM) for 24 hr. (**C**) Western blot analysis of AMPK phosphorylation and palmitate-induced impairment of IRS-1 and Akt phosphorylation in AMPK siRNA-transfected differentiated C2C12 cells treated with PDX (0–1 μM) for 24 hr. Human insulin (10 nM) was used to stimulate insulin signaling for 3 min. (**D**) Western blot analysis of AMPK phosphorylation in soleus muscle of mice treated HFD and PDX (five animals per treatment group). Means ± SEM were obtained from three separated experiments or five animals. ^***^ *P* < 0.001 and ^*^ *P* < 0.05 when compared to the control or the ND treatment. ^!!!^ *P* < 0.001, ^!!^ *P* < 0.01, and ^!^ *P* < 0.05 when compared to the palmitate or the HFD treatment. ^##^ *P* < 0.01 and ^#^ *P* < 0.05 when compared to the palmitate plus PDX treatment.

AMPK has been suggested to be an effective therapeutic target for insulin resistance and type 2 diabetes^14^ and was previously reported to be activated by PDX in skeletal muscle^5^. The current study showed that PDX augmented AMPK phosphorylation in a dose-dependent manner (Fig. 4A). As shown in Fig. 4B, palmitate stimulated NFkB nuclear translocation and IkBα phosphorylation. Suppression of AMPK by siRNA significantly abrogated the inhibitory effects of PDX on palmitate-induced inflammation (Fig. 4B). We next examined whether PDX-induced AMPK contributed to attenuation of palmitate-induced insulin resistance in differentiated C2C12 cells. Similar to the effects of PDX on inflammation, the suppressive effects of PDX on palmitate-induced impairment of insulin-stimulated IRS-1 and Akt phosphorylation were markedly abolished in the presence of AMPK siRNA (Fig. 4C). Furthermore, PDX administration significantly reversed HFD-suppressed AMPK phosphorylation in the soleus skeletal muscle of mice (Fig. 4D).

### PPARα is associated with the inhibitory effects of PDX on palmitate-induced inflammation and insulin resistance

**Figure 5:**
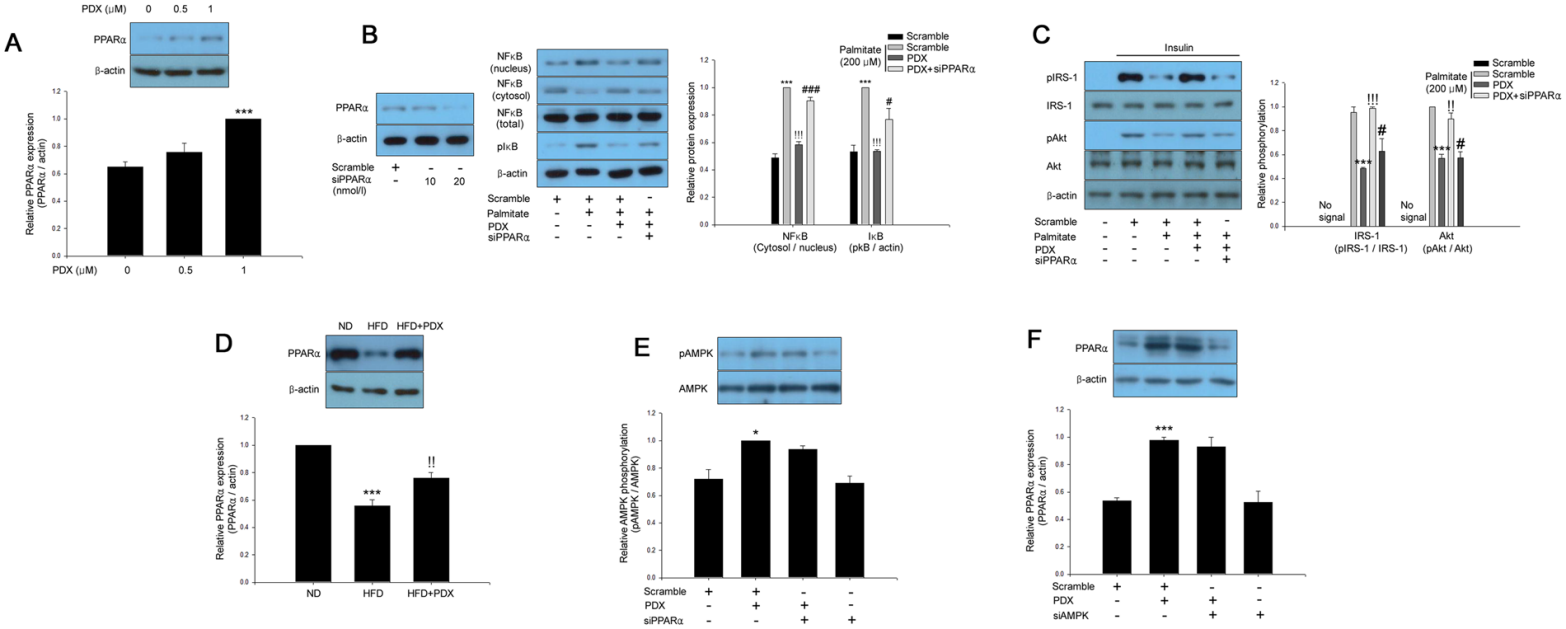
PDX ameliorates inflammation and insulin resistance through a PPARα-mediated pathway. (**A**) Western blot analysis of PPARα expression in differentiated C2C12 cells treated with PDX (0–1 μM) for 24 hr. (**B**) Confirmation of PPARα siRNA efficiency in differentiated C2C12 cells. Western blot analysis of palmitate (200 μM)-induced inflammation markers in PPARα siRNA (20 nM)-transfected differentiated C2C12 cells treated with PDX (0–1 μM) for 24 hr. (**C**) Western blot analysis of palmitate-induced impairment of IRS-1 and Akt phosphorylation in PPARα siRNA-transfected differentiated C2C12 cells treated with PDX (0–1 μM) for 24 hr. Human insulin (10 nM) was used to stimulate insulin signaling for 3 min. (**D**) Western blot analysis of PPARα expression in soleus muscle of mice treated HFD and PDX (five animals per treatment group). Western blot analysis of (**E**) AMPK phosphorylation in PPARα siRNA or (**F**) PPARα expression-transfected differentiated C2C12 cells treated with PDX (1 μM) for 24 hr. Means ± SEM were obtained from three separated experiments or five animals. ^***^ *P* < 0.001 and ^*^ *P* < 0.05 when compared to the control or the ND treatment. ^!!!^ *P* < 0.001 and ^!!^ *P* < 0.01 when compared to the palmitate or the HFD treatment. ^###^ *P* < 0.001 and ^#^ *P* < 0.05 when compared to the palmitate plus PDX treatment.

AMPK activates β-oxidation through a PPARα-dependent pathway^15^. Furthermore, activation of PPARα by fenofibrate attenuates inflammation^16^ and insulin resistance^17^. Therefore, we examined whether PDX increased PPARα expression in differentiated C2C12 cells. Treatment of differentiated C2C12 cells with PDX significantly induced PPARα expression in a dose-dependent manner (Fig. 5A). We also confirmed PPARα siRNA efficiency and showed that suppression of PPARα by siRNA markedly reduced the effect of PDX on palmitate-induced inflammation (Fig. 5B and C). These results suggest that PDX ameliorates palmitate-induced inflammation and insulin resistance through a PPARα-dependent pathway. Consistent with the *in vitro* results, PDX administration significantly increased PPARα expression in the soleus skeletal muscle of HFD-fed mice (Fig. 5D). PDX treatment increased AMPK phosphorylation, regardless of PPARα expression. Likewise, the induction of PPARα expression by PDX was not associated with AMPK (Fig. 5E and F).

### PDX induces β-oxidation through AMPK-PPARα-dependent pathways in differentiated C2C12 cells and skeletal muscle of HFD-fed mice

**Figure 6:**
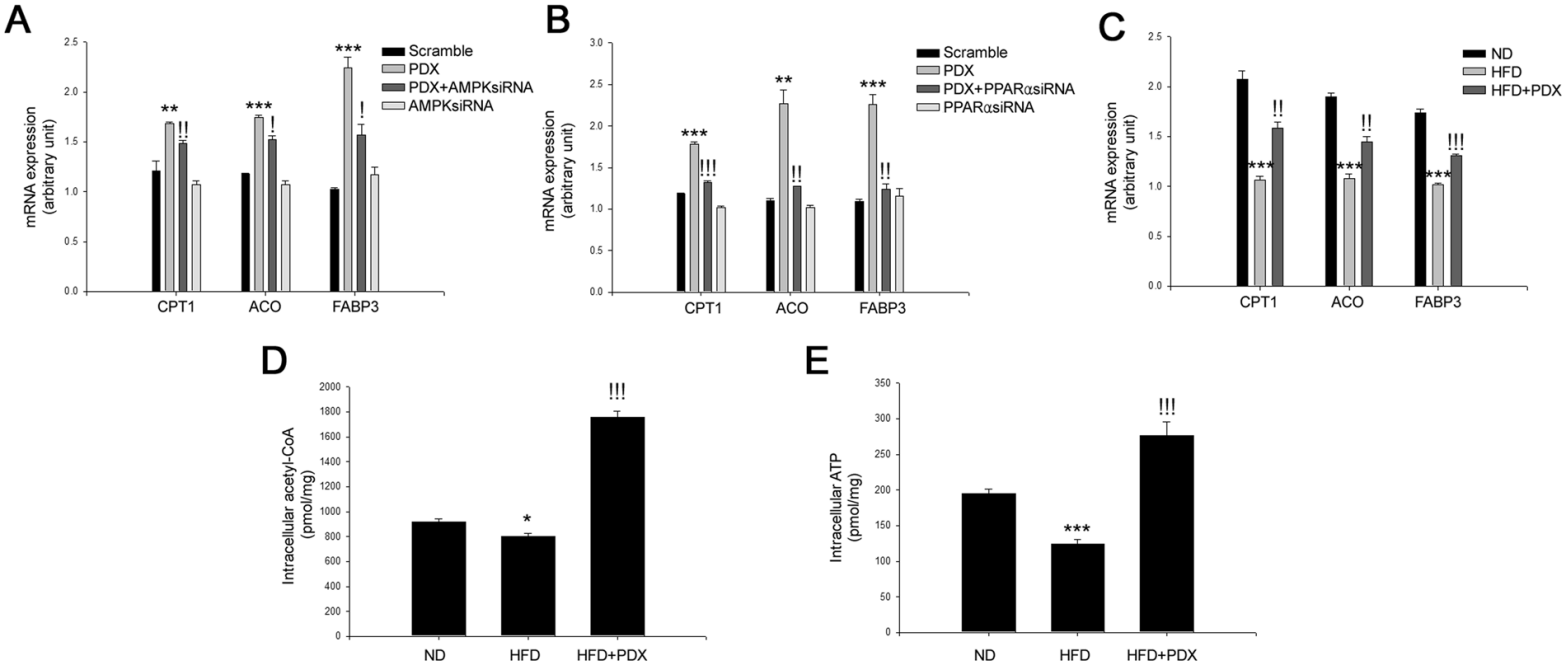
PDX stimulates fatty acid oxidation-associated gene expression. Quantitative real-time PCR analysis of CPT1, ACO, FABP3 mRNA expression in (**A**) AMPK (20 nM) or (**B**) PPARα siRNA (20 nM)-transfected C2C12 cells treated with PDX (1 μM) for 24 hr. (**C**) Quantitative real-time PCR analysis of CPT1, ACO, FABP3 mRNA expression in HFD-fed mice treated with PDX. (**D**) Intracellular acetyl-CoA and (**E**) ATP levels in soleus skeletal muscle of HFD-fed mice treated with PDX (1 μg/mouse/day) for 8 weeks (five animals per treatment group). Means ± SEM were obtained from three separated experiments or five animals. ^***^ *P* < 0.001 and ^**^ *P* < 0.01 when compared to the control or the ND treatment. ^!!!^ *P* < 0.001, ^!!^ *P* < 0.01, and ^!^ *P* < 0.05 when compared to the PDX or the HFD treatment.

Koves *et al*. reported that mitochondrial overload and incomplete fatty acid oxidation cause insulin resistance in skeletal muscle^18^. AMPK^15^ and PPARα^17, 19, 20^ have been reported to augment fatty acid oxidation. However, to the best of our knowledge, the effect of PDX on fatty acid oxidation has not been elucidated. Therefore, we next evaluated whether PDX-induced AMPK and PPARα could induce fatty acid oxidation. As shown in Fig. 6A and B, transfection with AMPK and PPARα siRNAs significantly abrogated the effect of PDX on expression of genes related to fatty acid oxidation, such as *CPT1*, *ACO*, and *FABP3* (Fig. 6A and B). In agreement with *in vitro* data, PDX administration significantly increased the mRNA expression levels of *CPT1*, *ACO*, and *FABP3* in the soleus skeletal muscle of HFD-fed mice (Fig. 6C). To confirm the stimulation of fatty acid oxidation by PDX, we also measured levels of acetyl-CoA and ATP, products of fatty acid oxidation. As expected, PDX administration markedly increased intracellular acetyl-CoA and ATP levels in soleus skeletal muscle of HFD-fed mice (Fig. 6D and E).

### AMPK and PPARα are not involved in the increase of muscle IL-6 expression by PDX

As PDX stimulates muscle IL-6 expression^5^, resulting in the suppression of hepatic gluconeogenesis, we examined whether PDX-induced AMPK or PPARα contribute to muscle IL-6 expression. Corresponding with a previous report^5^, treatment of differentiated C2C12 cells with PDX caused the induction of muscle IL-6 mRNA expression and secretion in a dose-dependent manner. However, AMPK or PPARα siRNAs did not abrogate the effect of PDX on IL-6 mRNA expression and secretion (Supplemental Figure S2A and B). In animal models, both IL-6 mRNA expression and serum IL-6 levels were increased by HFD. Furthermore, PDX administration further augmented IL-6 expression than the HFD group, showing that the higher serum IL-6 concentration in PDX-treated mice may be mainly derived from skeletal muscle (Supplemental Figure S2C and D).

## Discussion

Regular physical activity is an effective therapeutic approach for the treatment of insulin sensitivity and type 2 diabetes. During exercise, release of IL-6 from skeletal muscle into the bloodstream increases insulin sensitivity^21^. PDX mimics the effect of physical exercise by stimulating the secretion of IL-6 from skeletal muscle and thereby attenuates insulin resistance and hepatic gluconeogenesis. Therefore, White *et al*. suggested that PDX might be a novel agent for the treatment of diabetes^5^. However, the mechanisms by which PDX improves insulin resistance in skeletal muscle remain unclear.

AMPK is a key regulator of energy homeostasis and is known to ameliorate inflammation through inhibition of NFkB activity^22^. AMPK activation plays a beneficial role in metabolic disorders such as obesity and type 2 diabetes. Elevated levels of serum NEFA in conditions of obesity suppress AMPK activity^23^ and insulin sensitivity^24^. Obesity induces activation of NFkB and transcription of its target genes in liver^25^ and skeletal muscle^26^. In contrast, activation of AMPK suppresses inflammation by ameliorating endoplasmic reticulum stress and also reduces inflammation caused by reactive oxygen species through stimulation of thioredoxin^27, 28^. AMPK is activated in the muscle of both non-diabetic humans^29, 30^ and patients with type 2 diabetes^31^ during exercise. The current study demonstrated that PDX markedly induces AMPK phosphorylation, confirming previous observation^5^. Furthermore, suppression of AMPK abrogated the inhibitory effects of PDX on inflammation and insulin resistance, validating the role of AMPK in these effects. These results suggest that PDX attenuates hyperlipidemia-induced inflammation and insulin resistance *via* an AMPK-dependent pathway. We further investigated the relationship between PDX-induced muscle AMPK and IL-6, which is known to be largely responsible for attenuation of insulin resistance^5^. Suppression of AMPK did not affect IL-6 mRNA expression in and its secretion from C2C12 cells treated with PDX. These results suggest that IL-6 is not involved in the suppressive effect of PDX on hyperlipidemia-induced inflammation and insulin resistance through AMPK-dependent pathway in differentiated C2C12 cells. However, White *et al*. have reported that PDX-induce AMPK activation is not sufficient to attenuate insulin resistance in IL-6-knockout mice. We showed that PDX ameliorates insulin resistance *via* AMPK-dependent pathway in differentiated C2C12 cells. This may be due to insufficient activation of AMPK to improve insulin resistance by PDX administration in animal models or differences in between the *in vitro* and *in vivo* experimental conditions.

PPARα is considered a key metabolic regulator that may be associated with the positive effects of exercise in various tissues, such as skeletal muscle, liver, fat, and heart. Fenofibrate, a well-known agonist of PPARα, ameliorates the IL-1-induced inflammatory response in human aortic smooth muscle cells by inhibiting IL-6 expression^32^ and reduces plasma triglyceride with a concomitant increase in high-density lipoprotein-cholesterol in humans with metabolic syndrome^16^. Furthermore, fenofibrate shows protective effects against TNFα-induced inflammation *via* sirtuin1 (SIRT1)-associated inhibition of NFkB activity in endothelial cells^33^. Fatty acid oxidation and energy consumption resulting from PPARα activation in skeletal muscle and adipose tissue has many beneficial effects, including improved exercise endurance, insulin sensitivity, and reduced body weight^17, 19, 20^. Therefore, PPARα is an effective therapeutic target for the treatment of metabolic disease. The current study demonstrates for the first time that PDX treatment induces PPARα expression, leading to attenuation of inflammation through inhibition of the NFkB pathway.

We showed that induction of AMPK and PPARα by PDX resulted in suppression of inflammation in differentiated C2C12 cells. Several previous reports have demonstrated that NEFAs cause inflammation through NFkB activation and upregulation of its target genes, such as TNFα^34^. Concordant with previous reports, our results verified that palmitate induced nuclear translocation of NFkB and phosphorylation of IkBα and impairment of insulin-stimulated IRS-1 and Akt phosphorylation in differentiated C2C12 cells. In addition, knockdown of AMPK or PPARα significantly reduced the suppressive effects of PDX on palmitate-induced inflammation. Consistent with the effect on inflammation markers, PDX ameliorated palmitate-induced impairment of insulin-stimulated IRS-1 and Akt phosphorylation *via* AMPK and PPARα-mediated pathways. In *in vivo* experiments, PDX administration suppressed NFkB-mediated signal transduction and expression of proinflammatory cytokines, such as TNFα and MCP-1, in HFD-fed mice. These findings suggest that PDX simultaneously attenuates the inflammation and insulin resistance caused by palmitate or a HFD through AMPK and PPARα-associated pathways in skeletal muscle. Although NFkB activation in adipose tissue has been reported to be implicated as a crucial mechanism in the development of insulin resistance^35^, Hommelberg *et al*. have reported that NFkB activation is not necessary for palmitate-induced insulin resistance in skeletal muscle^36^. Therefore, the relationship between anti-inflammatory effect of PDX and its insulin-sensitizing effect requires further studies.

In this study, increased basal levels of plasma glucose were observed in HFD-fed mice by IPGTT and ITT; however, HFD-induced increase in basal plasma glucose levels was suppressed by PDX administration. This suppressive effect of PDX on basal glucose levels may result from AMPK- or PPARα-mediated improvement of insulin action or an effect on other signaling pathways including systemic muscle IL-6-mediated suppression of hepatic gluconeogenesis^5^.

Interestingly, PDX treatment resulted in body weight loss in HFD-fed mice despite no change in calorie intake. The decrease in body weight following PDX administration may be due to enhanced energy metabolism, including fatty acid oxidation leading to fat burning, because we confirmed that PDX administration stimulated AMPK phosphorylation and PPARα expression, which have a well-documented role in fatty acid oxidation^37–40^. Therefore, we further examined whether PDX induces β-oxidation in mouse skeletal muscle cells and found that genes associated with β-oxidation were upregulated in both differentiated C2C12 cells and mouse skeletal muscle. Naturally, fatty acid oxidation alone is not enough to suppress obesity^41^. In addition to fatty acid oxidation, lipogenesis and fat absorption also control obesity^42, 43^. Therefore, further studies are required to investigate the effects of PDX on lipogenesis, fat absorption and etc. in various organs. Moreover, since there is no difference in energy uptake, it is likely that there are possibilities by regulating other mechanisms such as amino acid and glucose metabolism for increase in energy expenditure. Thus, this also requires further investigations.

Intra-abdominal fat depots in obesity are closely tied to insulin resistance and type 2 diabetes^44^. Therefore, the effect of PDX on glucose and insulin tolerance may be linked to weight loss in animal models. In the present study, we further confirmed that PDX administration reduced the weight of liver and epididymal adipose tissue. These results suggest that PDX may reduce body weight gain from at least liver and visceral fat tissue and have a possibility to attenuate both glucose and insulin tolerance through weight loss. Further studies are therefore warranted in animals treated with lipid to induce acute insulin resistance without weight gain to determine whether weight loss is involved in the effect of PDX on both glucose and insulin tolerance. Additionally, we are preparing for clinical studies to investigate the relationship between PDX-induced weight loss and improvement of glucose and insulin tolerance.

In summary, the current study provides the first demonstration that PDX ameliorates palmitate- or HFD-induced insulin resistance and inflammation through an AMPK-PPARα-mediated pathway in skeletal muscle. Our report provides new insight into the mechanisms of PDX and the similarity of its effects of exercise and could lead to a therapeutic strategy for insulin resistance and type 2 diabetes.

## Methods

### Cell cultures, reagents and antibodies

The mouse skeletal muscle cell line C2C12 (ATCC, Manassas, VA, USA) was cultured in Dulbecco’s modified eagle medium (DMEM; Invitrogen, Carlsbad, CA, USA) supplemented with 10% fetal bovine serum (Invitrogen), 100 units/ml penicillin, and 100 μg/ml streptomycin (Invitrogen). Cells were cultured in a humidified atmosphere of 5% CO_2_ at 37 °C. Cells were supplemented with 2% horse serum to induce differentiation for 48 hr. C2C12 cells were confirmed to be free from mycoplasma. We used cells at passages 5–10 for all experiments. PDX (Cayman Chemical, Ann Arbor, MI, USA) was dissolved in ethanol. The final concentration of ethanol did not affect cell viability. Sodium palmitate (Sigma, St Louis, MO, USA) was conjugated to 2% BSA (fatty acid free grade; Sigma) dissolved in DMEM. In all experiments, cells were treated with palmitate-BSA for 24 hr and 2% BSA was used as a control. Cells were treated with 1 μM PDX and 200 μM palmitate for 24 hr without palmitate pretreatment or additional treatment steps. Cells were cultured in serum starvation media (without FBS) for 6 hr before insulin treatment. Insulin (10 nM) was used to stimulate insulin signaling, IRS-1 and Akt for 3 min after PDX and palmitate treatment. Anti-insulin receptor substrate (IRS)-1 (1:2,500), anti-phospho Akt (Ser473; 1:1,000), anti-Akt (1:1,000), anti-phospho AMPK (Thr172; 1:1,000), anti-AMPK (1:2,500), and anti-NFkBp65 (1:2,500), anti-phospho IkBα (Ser32; 1:1,000), and anti-PPARα (1:2,500) antibodies were purchased from Cell Signaling Technology (Beverly, MA, USA). Anti-phospho IRS-1 (Tyr632; 1:1,000) and anti-beta actin (1:5,000) were purchased from Santa Cruz Biotechnology (Santa Cruz, CA, USA).

### Animals, feeding, and treatment

This study was approved by the institutional animal review board of the Institutional Animal Care and Use Committee of Korea University, Seoul, Korea. Animal studies were conducted in accordance with the Guide for the Care and Use of Laboratory Animals (NIH publication, 8th edition, 2011). A control group and two experimental groups of 8-week-old male C57BL/6J (B6) mice were given a normal diet (ND; Brogaarden, Gentofte, Denmark) or a high-fat diet (HFD; Research Diets, New Brunswick, NJ, USA) for 8 weeks. The HFD plus PDX group were additionally administered PDX intraperitoneally (1 μg/mouse/day) and the ND and HFD groups were also administered vehicle intraperitoneally with same volume (mouse/day) for 8 weeks. Mouse soleus skeletal muscle samples were isolated 10 min after treatment of the mice with human insulin (Novo Nordisk, Princeton, NJ, USA; 10 U/kg body weight) *via* intraperitoneal injection. To perform the intraperitoneal glucose tolerance test (IPGTT), mice were fasted for 12 hr (overnight) and then intraperitoneally injected with glucose (2 g/kg body weight). Serum glucose levels were measured at glucose challenge and 30, 60, 90, and 120 min thereafter. To perform the insulin tolerance test (ITT), mice were fasted for 6 hr and then given an intraperitoneal injection of human insulin (1 U/kg body weight). Serum glucose levels were measured at glucose challenge and 15, 30, 45, and 60 min thereafter. IPGTT was performed three days before sacrifice of mice treated with HFD and PDX for 8 weeks. One day after the end of IPGTT, ITT was performed. Serum glucose levels were measured using Accu-Check III glucose analyzer. Upon completion of the study period, all experimental mice were sacrificed under anesthesia after an overnight (12-hr) fast.

### RNA extraction and quantitative real-time PCR

Total RNAs from harvested C2C12 cells and soleus skeletal muscle tissue were isolated using TRIzol reagent (Invitrogen, Carlsbad, CA). Gene expression was measured by quantitative real-time PCR (qPCR) using the fluorescent TaqMan 5′nuclease assay on an Applied Biosystems 7000 sequence detection system (Foster City, CA, USA). qPCR was performed using cDNA with 2× TaqMan Master Mix and the 20× premade TaqMan gene expression assays (Applied Biosystems). qPCR conditions were 95 °C for 10 min, followed by 40 cycles of 95 °C for 15 s and 60 °C for 1 min. Expression of mouse carnitine palmitoyltransferase 1 (*CPT1*) (Mm00463960_ml; Applied Biosystems), acyl-CoA oxidase (*ACO*) (Mm00801417_ml), and fatty acid binding protein 3 (*FABP3*) (Mm02342495_ml) mRNA was normalized to that of mouse beta-actin (Mm00607939_sl; Applied Biosystems).

### Western blot analysis

Differentiated C2C12 cells were harvested and proteins were extracted with lysis buffer (PRO-PREP; Intron Biotechnology, Seoul Korea) for 60 min at 4 °C. Protein samples (35 μg) were subjected to 12% SDS-PAGE, transferred to a nitrocellulose membrane (Amersham Bioscience, Westborough, MA, USA), and probed with the indicated primary antibody followed by secondary antibody conjugated with horseradish peroxidase (Santa Cruz Biotechnology). The signals were detected using enhanced chemoluminescence (ECL) kits (Amersham Bioscience).

### Enzyme linked immunosorbent assay (ELISA)

Mouse serum TNFα and MCP-1 were measured with each ELISA kit (R&D Systems, Minneapolis, MN, USA) according to the manufacturer’s instructions.

### Transfection with siRNAs for gene silencing

Small interfering (si) RNA oligonucleotides (20 nM) specific for AMPK and PPARα were purchased from Santa Cruz Biotechnology. To suppress gene expression, cell transfection was performed with Lipofectamine 2000 (Invitrogen) in accordance with the manufacturer’s instructions. In brief, the cells were grown to 50% to 60% confluence and fully differentiated, followed by serum starvation for 6 hr. The cells were transfected with validated siRNA or scramble siRNA at a final concentration of 20 nM in the presence of transfection reagent. After transfection, the cells were harvested at 36 hr for protein extraction or further treated with 10 nM insulin, 1 μM PDX, or 200 μM palmitate.

### Cell fractionation for measurement of NFkB nuclear translocation

Cells were fractionated using a Nuclear/Cytosol Fractionation Kit according to the manufacturer’s instructions (Bioviosion, Milpitas, CA, USA).

### Measurement of glucose uptake and acetyl-CoA and ATP content

Glucose uptake levels were measured using Glucose Uptake Assay Kit^TM^ (Abcam, Cambridge, MA, USA). In brief, proliferating and differentiating C2C12 cells were seeded at 5 × 10^4^ cells/well in black walled/clear bottom 96-well plates (Corning, Inc., Corning, NY, USA) in DMEM containing 10% FBS. Upon reaching a confluency of 50%, differentiation was induced with differentiating media consisting of high glucose DMEM, 2% horse serum. After 48 h, media was changed to media containing palmitate (250 μM) or PDX (0–1 μM) for 18 h. Following treatment, media was removed from wells and treated with 10 nM insulin and 1 mM 2-Deoxyglucose (2-DG) for 30 min. Afterwards plates were centrifuged for 1 min at 500 rpm and incubated for 1 hr at room temperature. After 2-DG taken up by the cells were extracted by extraction buffer in kit, 2-DG uptake levels were then measured at a wavelength of OD 412 nm on a BioTek Synergy HT plate reader (BioTek Instruments, Inc., Winooski, VT, USA). Intracellular levels of acetyl-CoA were measured using PicoProbe Acetyl CoA Assay Kit^TM^ (Abcam) and intracellular ATP levels were measured using ATP Assay Kit^TM^ (Abcam) in differentiated C2C12 cells or soleus skeletal muscle were measured according to the manufacturer’s instructions.

### Statistical analysis

All analyses were performed using the SPSS/PC statistical program (version 12.0 for Windows; SPSS, Chicago, IL, USA). Results are presented as the fold of the highest values (means ± SEM). All *in vitro* experiments were performed at least three times. Student’s t test or two-way ANOVA were used for statistical analysis.

## Electronic supplementary material

supplemental figures_legends
