# Supplementary material for: Protectin DX ameliorates palmitate- or high-fat diet-induced insulin resistance and inflammation through an AMPK-PPARα-dependent pathway in mice
Source: Sci Rep. 2017 May 3;7:1397. doi: 10.1038/s41598-017-01603-9 (PMC5431091; doi:10.1038/s41598-017-01603-9)

Protectin DX ameliorates palmitate- or high-fat diet-induced insulin resistance and inflammation through an AMPK-PPARα-dependent pathway in mice

Tae Woo Jung[1], Hyung-Chun Kim[2], A.M. Abd El-Aty[3, 4], Ji Hoon Jeong[5*]

Running title: PDX improves insulin resistance in myocytes

[1]Research Administration Team, Seoul National University Bundang Hospital, Gyeonggi, Republic of Korea

[2]Neuropsychopharmacology and Toxicology Program, College of Pharmacy, Kangwon National University, Chunchon, Republic of Korea

[3]Department of Veterinary Pharmacology and Toxicology, College of Veterinary Medicine, Konkuk University, Seoul, Republic of Korea

[4]Faculty of Veterinary Medicine, Cairo University, 12211-Giza, Egypt

[5]Department of Pharmacology, College of Medicine, Chung-Ang University, Seoul, Republic of Korea

[*]Corresponding author: Ji Hoon Jeong, Professor, PhD

Department of Pharmacology, College of Medicine, Chung-Ang University, 221, Heuksuk-dong, Dongjak-gu, Seoul 156-756, Korea. Tel: +82-2-820-5688, Fax: +82-2-826-5680, E-mail: jhjeong3@cau.ac.kr

Supplemental Figure S1. **PDX administration reduces the weight of epididymal fat tissue and liver**. Measurement of epididymal fat tissue (A) and liver (B) weight in mice (five animals per treatment group). Means ± SEM were calculated data obtained from five separated animals. $^{***}P<0.001$ when compared to the ND treatment. $^{!!!}P<0.001$ and $^{!}P<0.05$ when compared to the HFD.

Supplemental Figure S2. **AMPK and PPARα are not involved in the increase of muscle IL-6 expression by PDX.** (A) Quantitative real-time PCR analysis of IL-6 mRNA expression and (B) ELISA of IL-6 secretion in AMPK (20 nM) or PPARα siRNA (20 nM)-transfected C2C12 cells treated with PDX (0-1 μM) for 24 hr. (C) Quantitative real-time PCR analysis of IL-6 mRNA expression in soleus skeletal muscle of HFD-fed mice treated with PDX (1 μg/mouse/day) for 8 weeks (five animals per treatment group). (D) ELISA of serum IL-6 in HFD-fed mice treated with PDX (five animals per treatment group). Means ± SEM were obtained from three separated experiments or five animals. $^{*}P<0.05$ and $^{**}P<0.01$ when compared to the control or the ND treatment. $^{!!!}P<0.001$ when compared to the HFD treatment.

Supplemental Figure S1

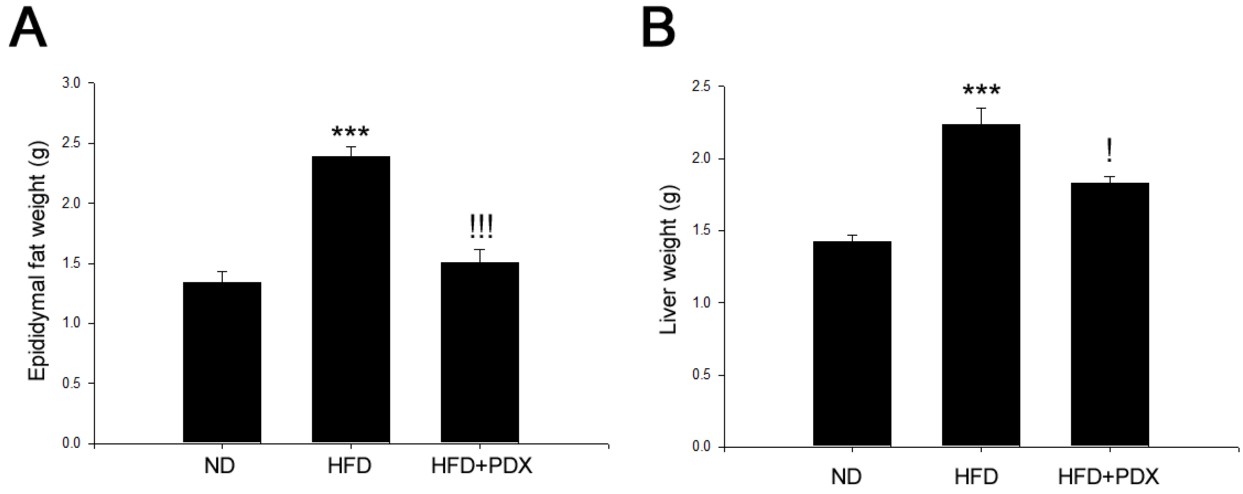

Supplemental Figure S2

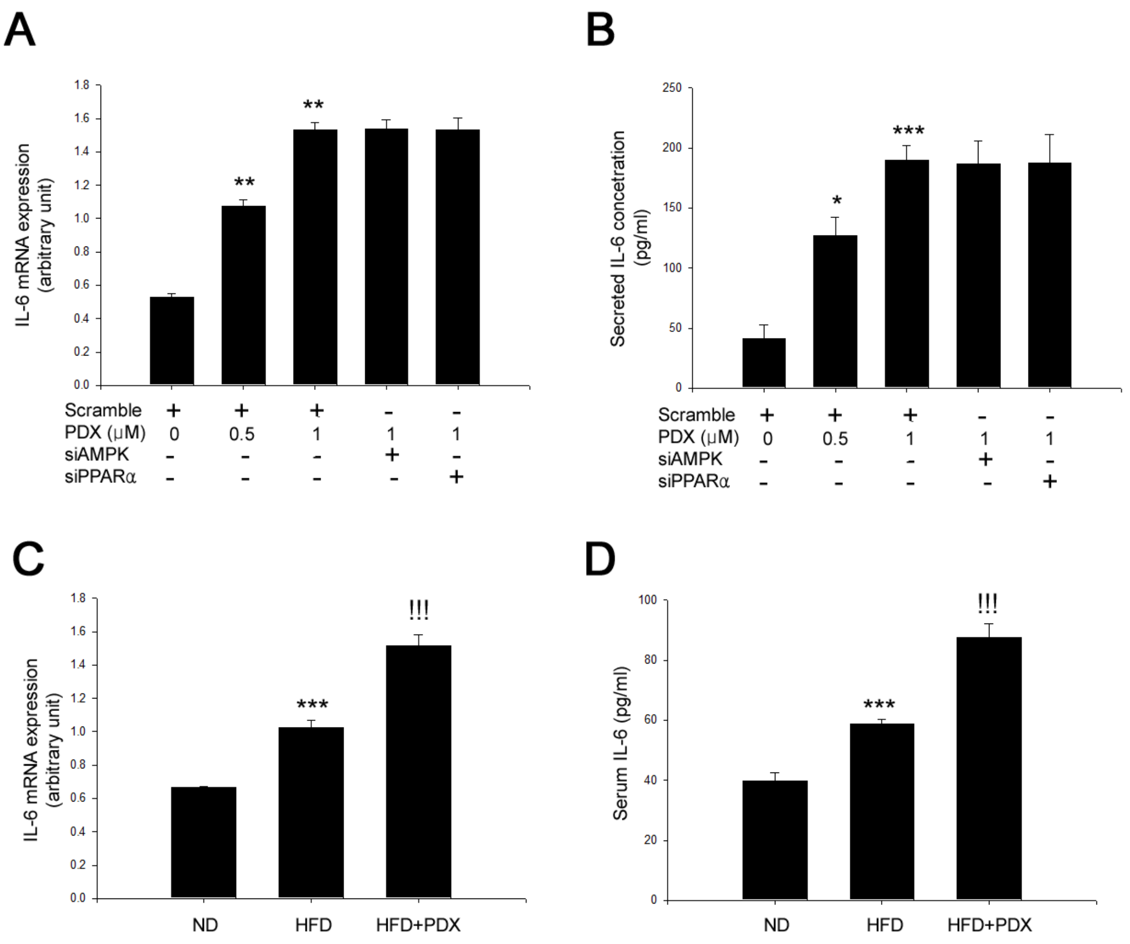

Supplement: Supplementary file 1 — supplemental figures_legends [file 41598_2017_1603_MOESM1_ESM.pdf]
